# Addressing violence against children: A case review in the state of Qatar

**DOI:** 10.3389/fpubh.2022.859325

**Published:** 2022-12-06

**Authors:** Abdulla Saeed Al-Mohannadi, Sanaa Al-Harahsheh, Sajeda Atari, Nadeem Jilani, Ghalya Al-Hail, Kennedy Sigodo

**Affiliations:** ^1^World Innovation Summit for Health (WISH), Qatar Foundation, Doha, Qatar; ^2^UNICEF, Doha, Qatar; ^3^Sidra Child Advocacy Program, Department of Emergency Medicine, Sidra Medicine, Doha, Qatar; ^4^Department of Nursing and Community Health, Glasgow Caledonian University, London, United Kingdom

**Keywords:** Child health, Child Abuse, public health, violence against children, thematic analysis, socioecological model

## Abstract

**Introduction:**

Violence against children (VAC) is a critical public health issue that affects billions of children worldwide. The combination of its prevalence and severity of effects on children creates an urgent need for effective interventions. Multiple studies associate VAC with lifelong implications that affect children through adulthood. In Qatar, multiple approaches such as legislation are being used to protect children from all forms of violence. Despite the gravity of the issue, there is still low readiness for the prevention of VAC in Qatar. This review aimed to map approaches to addressing VAC in Qatar from the panelists' perspectives on current approaches to addressing VAC.

**Methods:**

The review obtained data from a recorded video entitled “A Public Health Approach to Addressing Violence Against Children.” The panel discussion in this video clip was organized as a side event of the WISH virtual summit by UNICEF and WISH on World Children's Day, held in Qatar in November 2020. The video was transcribed and analyzed using thematic analysis.

**Findings:**

It shows the importance of both global and national level interventions in addressing VAC. The review uses the socioecological model to show relationships among different levels of interventions addressing VAC in Qatar. The findings highlight the national approaches to addressing VAC using public health, and legislative and policy approaches.

**Discussion:**

The interventions addressing VAC at different levels in Qatar are interconnected. Delineating each level is key to the formation of holistic interventions that leverage global, regional, national, communal, familial, and individual factors that support interventions to address VAC.

## Introduction

Article 19 of the Convention of the Rights of the Child (1) defines violence as, “all forms of physical or mental violence, injury, and abuse, neglect or negligent treatment, maltreatment or exploitation, including sexual abuse”. The types of violence afflicting children vary widely and include maltreatment, bullying, youth violence, intimate partner violence, sexual violence, and emotional or psychological violence (2). VAC is a global problem occurring not only in Qatar but in multiple other countries with long-term impacts on the growth and development of children that persist into adulthood (3).

The combination of its prevalence and adverse effects on children make VAC a critical issue requiring urgent and effective interventions (4). Approximately one billion children experience violence annually around the world (5). This represents half of the world's children, which translates to one out of two children between 2 and 17 years being afflicted by a form of violence each year (6). A child who is abused, or experiences different types of violence, is more likely to abuse or get abused by others as an adult—leading to a vicious cycle of violence (7, 8). Children who experience higher levels of exposure to adverse childhood events, such as emotional, sexual, psychological and physical abuse, neglect, parental conflict, divorce, parental alcoholism and substance abuse, are at higher risks of developmental problems, mental illnesses, cardiovascular diseases, behavioral problems, cognitive disorders, and some types of cancers (9, 10).

Global recognition of the enormity and scale of VAC on the economy and society has seen the drafting of the Convention on the Rights of the Child and the Sustainable Development Goals (SDGs) to rally nations to commit to ending all forms of VAC by 2030 and increase the investment in violence prevention and response. There are national mechanisms and institutions in Qatar that ensure the survival, safety, development, protection of children from all forms of violence, and abuse. In Qatar, the constitution and laws guarantee every child the right to health care, education, and protection from all forms of VAC and abuse. The WHO, UNICEF, and a consortium of international agencies worked together to develop an evidence-based technical package of seven key strategies to end violence against children. The seven key strategies are represented by the acronym INSPIRE and encompass “implementation and enforcement of laws; norms and values; safe environments; parent and caregiver support; income and economic strengthening; response and support services; and education and life skills” (11). INSPIRE has been vital in supporting national strategies to eliminate VAC (11).

Despite recognizing the enormity and the concerted efforts to end VAC, the efforts still face multiple hurdles in attaining national and global goals. The major challenge is the lack of reporting and documenting of the VAC within countries. The dearth of data across countries is a testament to the inadequate and incomplete systems within national frameworks for eliminating VAC. The data challenge is double, orchestrated by both absence of effective data collection frameworks and the absence of standardized measures of different forms of VAC (12). Arguably, the lack of information downplays the extent of VAC by portraying it as a marginal problem that only affects a small category of children and that is perpetrated by a specific group of people in the community. Also, the seeming lack of evidence orchestrated by the lack of data negates the urgency of addressing VAC by portraying it as less important.

The World Report on Violence Against Children advocates for the collection and reporting of national data on maltreatment and VAC (13). According to a review conducted by Pinheiro (13), the data collection tools used to evaluate and measure VAC are problematic due to myriads of issues. For instance, they are not standardized as they use different definitions, selection of samples and inter-country variations in measurement methods (14). The World Report (13) recommends setting standards for instruments that could assess epidemiology and services for children. Efforts have been geared toward addressing these inadequacies. For instance, the establishment of the Child Protection Monitoring and Evaluation Reference Group (CP MERG) in 2010 enabled the development of outputs that support countries and other organizations to collect reliable and valuable data. Similar calls led to the development of three Child Abuse Screening Tools (ICASTs) for parents, youths between 18 and 21, and children between 11 and 18 years by the International Society for Prevention of Child Abuse and Neglect (ISPCAN) (14). These tools have found application in research and have been instrumental in exploring childhood maltreatment in Qatar (3).

In Qatar, various laws have been enacted to ensure that children are being protected from all forms of violence. Such include, but are not limited to Articles 22 and 269, from Qatar Law No. 11 of 2004 on Issuing the Penal Code, that strive to protect the child from any form of exploitation they may experience be it in the form of “physical, mental or spiritual neglect” (15, 16). Although children's rights are an integral part of the 2030 National Strategy and considered pivotal in guaranteeing the health and security of the children in Qatar, there still is much to be done in terms of infrastructure and mechanisms that will ensure that the children are being protected from any form of violence they may encounter (17). On a more promising note, the country is finalizing its protection legislation on the rights of the child (17). UNICEF reported that the state of Qatar is amongst the countries rating low in the Middle East and Northern Africa (MENA) Region with regards to corporal punishment (17). Despite the efforts of various organizations in the country to tackle this ongoing issue, what is lacking that can make all the difference is a “child-centered approach” that puts the interest of the child at the forefront of its agenda (17). One of the main struggles that children face not only in Qatar but in the MENA Region is the lack of clear mechanisms of complaint when a child experiences some form of violence (17). This difficulty is attributed to the taboo nature of the discourse on the subject and children's dependence on their kin for relevant support. On a conclusive note, despite the many efforts of institutions be it from the Protection and Social Rehabilitation Center (AMAN), Family Consulting Center (Wifaq), Hamad Medical Corporation (HMC), or National Ministries, the State is in the process of forming its very own Child Law and establishing a National Plan that pushes for action in this area with the aid of key stakeholders.

Extant evidence shows that VAC is an important public health issue in Qatar that requires urgent and effective interventions (18). Despite the gravity of the issue, there is still low readiness for the prevention of VAC in Qatar (18). It is important that the existing approaches used to address VAC be mapped against global wide-ranging prevention strategies that have proved effective. This review aimed to map approaches to addressing VAC in Qatar against the panelists' perspectives on current global approaches to addressing VAC.

## Materials and methods

### Data collection

The review obtained data from a recorded video entitled “A Public Health Approach to Addressing Violence Against Children”. The panel discussion in this video clip was organized as a side event of the World Innovation Summit for Health (WISH) virtual conference by UNICEF and WISH on WORLD CHILDREN'S DAY, held in Qatar in November 2020. The video was subsequently published on YouTube by WISH as an official video from the event.

The video provided an ideal setting for analyzing the wide-ranging discussion since the panel consisted of persons from disparate backgrounds. The diverse panel enabled a comprehensive and rich discussion informed by varied areas of expertise. The discussion panel comprised six speakers: a moderator and five panelists affiliated with different global and national organizations. The moderator asked each panelist a question in turn; therefore, the form of the panel discussion was more similar to a group interview than a debate. The moderator asked a different but related question while effectively connecting the question to each panelist's expertise and background. To achieve the aim of the review, a qualitative thematic analysis was used to analyze the plenary session.

### Recruitment of panelists

The panelists who took part in the discussion were selected by the organizers of the event (i.e., WISH and UNICEF) based on their area of expertise not by the authors of this paper. Characteristics of the panelists are summarized in Table 1.

**Table 1:** Characteristics of panelists.

**Participants in the panel discussion**	**Title**	**Organizational affiliation**	**Level of organization**
Moderator	Presenter and producer of “women voices” or “bekasretta”	Al Jazeera Arabic Channel	Global
Panelist 1	Special Representative	United Nations Special Representative of the Secretary General on violence against children	Global
Panelist 2	Regional advisor	UNICEF regional advisor on child protection	Regional: Middle East and North Africa
Panelist 3	Director of international programs and research	Coram Children's Legal Center	Global
Panelist 4	Director of family affairs	Ministry of Administrative Development, Labor and Social Affairs in Qatar	National
Panelist 5	Executive director	Dreama and Aman Centers for Qatar Social Work Foundation	National

### Ethical consideration

Ethics approval was not required for this review as it was based on a video that was consensually recorded, publicly posted and the content made available and accessible to any internet user. Accessing the recorded video did not require any interaction with the panelists. Nonetheless, permission was obtained from the organization (WISH center) that recorded and published the video online to allow analysis and use for research purposes.

### Data analysis

In the video used in this review, English and Arabic were spoken in the session and Arabic interventions were translated into English. Although simultaneous interpretation services were provided to the panelists and the audience at the venue, only the English and the translated interventions are available in the video. The video clip of the high-profile panel discussion was transcribed by a researcher from the team and reviewed by a second researcher. A thematic analysis approach (19) was utilized for qualitative analysis of the transcribed data. The process began with familiarization with the plenary session's transcript. Familiarization was achieved through repeated reading. Two researchers (KS and AA) read the transcriptions and generated proposed codes. The team then met to review proposed codes and worked jointly to create a final consolidated coding structure (Table 1). This coding structure was then reviewed by the lead members of the Qatar team (AA) to provide validation from their local and cultural proficiency that the coding structure was appropriate. Once the transcribed data was completely coded, the researchers (KS and AA) reviewed the coded transcripts and worked collaboratively to identify themes, utilizing an inductive approach. Any inconsistencies were reviewed by the authors KA and AA and clarified.

Thematic analysis provided the flexibility that is associated with the epistemology of qualitative research. The need to incorporate the panelists' perspectives and the meaning they attach to the various aspects of VAC. When considering the contextual variations on the perspectives on VAC (20), it may be argued that the “contextualist” approach is in essence constructionism. Therefore, it was essential for the researchers to take into account the context in which the discussion that was recorded took place.

Context refers to “the set of circumstances surrounding an event or situation that help in its interpretation” (21, 22). As has been argued by Knoblauch and Schnettler (23), analyzing a video does not merely consist of “content” analysis of its dialogue, but rather systematic analysis of the “context” in which interactions among participants are embedded. A speaker's actions and responses are shaped by the given context, and a sequence of action can only be understood with reference to the context (24). In fact, a public video provides additional information, such as the layout of the venue in which interactions take place and the seating arrangement of the speakers, which may influence the flow of discussion. It must be noted that when a public discussion is videotaped, the sequence of discussion might be outlined in advance in a written agenda. Hence, dynamic interactions can be limited in a well-organized event as compared to video footage of a direct field interview or a conversation between strangers, which are likely to take place with a wide range of contingencies (25). However, as argued by Deppermann et al. (26) that interaction and its transition in a formal event are still influenced by an actual performance, and that participants often collectively allow some divergence to emerge. Additionally, sophisticated, and high-level speakers often express their views by spontaneously relating or comparing with other panelists' opinions. It is, therefore, very important to be attentive to interactions in a real-time context, since interactions will unfold from the beginning to the end of a video (22, 27).

**Figure 1:**
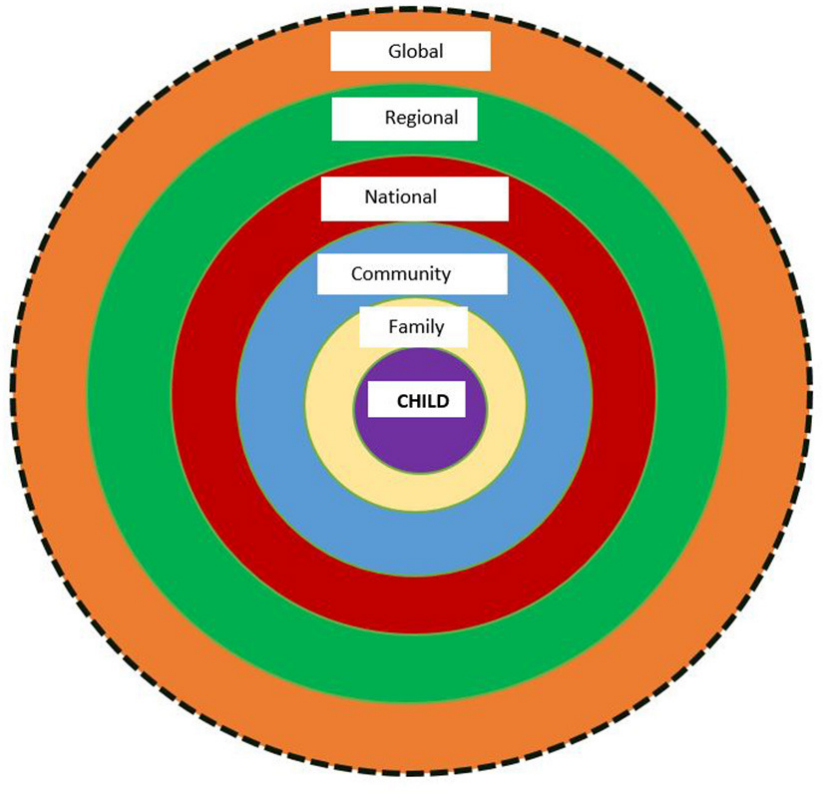
Mapping different levels of interventions to address VAC in Qatar (Adapted from Kelly et al. (28)).

Adapted Bronfenbrenner's socioecological model by Kelly et al. (28) was used to map the VAC prevention approaches. The adapted model recognizes the influence of different levels of socioecological factors on VAC and the relationship between the different levels of interventions. Most important, the model has the child at its core, hence, suits the review's “child-centered approach” that puts the interest of the child at the forefront of its VAC agenda. The adapted socioecological model divided the intervention levels into five levels with the child at the center as shown in the Figure 1 below. In this review, the revised socioecological model includes different levels of interventions to address VAC in Qatar. The model starts with the distal global, regional (Middle East and North African Region) and national level interventions and moves to the more proximal interventions within the community, family and child levels. The adapted model included a dotted global ring of influence that corresponds with the need for national or local contextualization of global VAC interventions (28).

## Findings

The findings from the thematic analysis of the panel discussion identified public health and, legislation and policy as the two main approaches to addressing VAC in Qatar. Adapted socioecological model indicates the different levels at which the two main approaches are utilized and the type of interventions involved. The summary of findings based on the model is presented in Table 2.

**Table 2:** Summary findings of levels of VAC interventions using adapted socioecological model.

**Model**	**Level**	**Findings summary**
Socioecological	Global	• Global guidelines and frameworks support regional and national level interventions. • Global data enable estimations and inter-country comparison of efforts in addressing VAC.
	Regional	• MENA region has shared culture, religion and family structures. • Co-operation and collaboration among the countries within the region is crucial for addressing VAC in constituent countries.
	National	• Need to contextualize interventions to suit the Qatari context. • Inadequate planning and slow policymaking hampering efforts.
	Community	• Interventions aim to build a safe, nurturing, empowering and protective environment. • Success of programs contingent on working with the community. Community as the point to create the demand and acceptance of services.
	Family	• Family is the context within which much violence against children occur. • There is less understanding of family dynamics and psychology of abuse within the families. • Closed families are resistant to legislations that they consider as intrusion into family affairs.
	Child	• Children have agency and should be involved in the interventions that affect them.

### Public health approaches

Panelists identified public health approaches as key in addressing VAC. Multisectoral approach to primary, secondary, and tertiary levels of prevention was cited by some panelists as captured by the excerpt below.

“The public health approach provides a useful framework for both continuing to investigate and understand the causes and consequences of violence and for preventing violence from occurring through programs, policy interventions and advocacy, public support goals, or a science base that is extraordinary and cross-sectional, including health, education, social services, justice policy and the private sector.”

(Panelist 1- UN Special Representative)

Another panelist drew an example from Qatar and highlighted some of the organizations and government ministries involved in addressing VAC:

“…when we are speaking about child protection, it has to be really a national commitment within the top level. This is important. The Minister of Labor and Social Affairs has to coordinate to work on it. Sure. Because we need to involve Minister of Justice, Minister of Education, all the Ministries and Minister of Finance, because this is important…”

(Panelist 1—UN Special Representative)

The core insight of this theme is focusing on promoting health and wellbeing of children as a means to preventing VAC.

### Legislative and policy approaches

Despite legislation and policy interventions being lauded for the potential of addressing VAC. Panelists cited gaps in implementation and evaluation as challenges hindering realization of intended outcomes. A panelist stated:

“So we know that we need laws and regulatory frameworks and policies and that I think the international bodies have been good at. But we also need effective governance structures in countries.”

(Panelist 3—Director of a Children's Legal Center) “What we need is to make legislation implementable. And XX already mentioned that you can have the most beautiful legislation, the most perfect piece of law in the world, if you do not have social workers to respond to the needs of children. If teachers in school do not want to contribute to keeping children safe, parent behaviors do not change. It doesn't contribute to any significant change”

(Panelist 2—UNICEF Child protection regional advisor for MENA)

Policies and legislations need to be flexible and fast adapting to contain emerging issues. The policy on cyberbullying in Qatar was described by a panelist as slow to develop and that the emergence of new threats posed by developments in the cyberspace called for faster legislations.

### Levels of interventions according to the socioecological model

Socioecological model was seen by the panelists as a sustainable and acceptable approach to addressing VAC. One of the panelists stated that:

“…use of a level socioecological model to better understand violence and the effect of potential prevention strategies. This model considers the complex interplay between individual relationship, community and social factors. This approach is more likely to sustain prevention efforts over time than any single intervention in adopting the public health approach.”

(Panelist 1—UN Special Representative)

Socioecological model showed the role played by children's social environments in the perpetuation of violence through a vicious cycle of inter-generational and societal transmission of violence. Thereby, providing a framework indicating different levels interventions needed to take action at different levels of influence. Collaborations across all levels were deemed necessary for any meaningful change to occur. One panelist stated:

“…any real change still requires some time, but mostly requires collaborative efforts from this family itself, from the governments, from the legislative part.”

(Panelist 4—Director of the Family Affairs Department)

These are summarized under the global, regional, national, community, family, and child level interventions.

#### Global level

VAC is a multifaceted problem and occurs in different forms—affecting children all over the world. As a universal issue, there are global interventions that are spearheading the elimination or reduction of VAC. Some of the organizations mentioned in the plenary included UNICEF and UNESCO. United Nation's Global agenda such as the 2030 agenda—Sustainable Development Goals was mentioned as part of the global efforts in addressing VAC.

The global guidelines and frameworks to eliminate VAC formed a solid base to develop effective interventions at a national and regional level. Similarly, global data on prevalence and interventions provide a crude estimate of the magnitude of VAC in the world and establishes a benchmark for countries to gauge their efforts in addressing VAC against other nations—leading to its prioritization in regional and national dialogues. One panelist mentioned:

“protection from violence is gaining reasonable recognition on international, regional, and national agendas leading to the development of national plans, you got reports that many, many challenges remain. Global status report on preventing VAC found that law against violence affecting children are widely enacted.”

(Panelist 1—UN Special Representative)

#### Regional level

Region referred to the Middle East and North Africa region. Other panelists used the term Arab region and Gulf regions. Region level interventions were seen as key due to shared culture, religion and family structures within the countries. Therefore, co-operation and collaboration among the countries within the region were seen as crucial in changing the pre-dominant passive view of children among families as well as regional sharing of information on best practice. An intervention working well in one of the countries would need little to no modifications to be replicated in another country due to the aforementioned shared attributes. A regional adviser stated:

“And I'm hopeful that in the years to come, we will create a broader and sustainable cooperation in the Arab region. We have a lot to learn across the region, from North Africa to the Middle East and many new practices emerging. So I would say if we are able to bring all this knowledge in the Arab region and to really learn from each other, that would be definitely be an accelerator.”

(Panelist 2—UNICEF Child protection regional advisor for MENA)

#### National level

Contextualization of the interventions was cited as key to the success of each of the interventions. Just because an intervention had registered success in one country, was not a guarantee that it would work in another. This was due to the variations in the contexts such as religion, culture, demographic composition, values, and traditions of a given country. Other variations in context include risk factors children are exposed to, the prevalence of certain forms of VAC and the national frameworks available to support the implementation and evaluation of the globally steered agenda on VAC. One panelist stated:

“I cannot say that the solution will work in Qatar and it will work elsewhere in the world. I think that every society has its own, let's say, way of solving the problems according to the norms and social services that they have.”

(Panelist 5—Executive director of a National Foundation)

Despite the efforts at the national level in most countries, there are still lapses that need to be addressed. When commenting on an undisclosed report, one of the panelists stated:

“Many countries have some ideas to support national violence prevention, but few have plans to expand and include measurable targets. The report found also that 89% of countries allocate responsibility to multiple sectors addressing violence against children with education, health, justice, and sectors most often mentioned. And 80 percent of countries reported having at least one national action plan to prevent violence against children. This promising finding contrasts with the fact that just one-fifth of the countries reported that their national action plans are fully funded.”

(Panelist 1—UN Special Representative)

The community constituted the proximal social environment to children and was viewed as imperative for the success of any intervention has to buy in to child protection. Community-level interventions were seen as those aiming to build a safe, nurturing, empowering and protective environment. These were deemed only achievable when working with the community. The community was also seen as the point to create the demand of services ensuring that interventions designed to address VAC are demanded and accepted by communities. A panelist reiterated this by stating that

“And it's not enough to have services available is how do we reach out to communities to request those services? So that would be a first important step.”

(Panelist 2—UNICEF child protection regional advisor for MENA)

#### Family level

Interventions aimed at supporting families and providing alternative family care were seen as necessary for addressing VAC in children. Family was especially important for three main reasons: Firstly, it was the context within which much VAC occurred. Secondly, there was less understanding of family dynamics and the psychology of abuse within the families. Thirdly, within the Arab society, families tend to be closed units that are more often than not resistant to any legislations that are considered as intrusion into the affairs of the family. Understanding the resistance as well as resilience within families was mentioned as key to ensuring collaborations at the family level. According to one of the panelists.

“We need to be able to support families and help them to use positive discipline rather than physical discipline. We need services for children who cannot stay with their families because the violence is just too bad.”

(Panelist 3—Director of a Children's Legal Center)

#### Child level interventions

Child participation emerged as a less explored but useful aspect in addressing VAC. According to some of the panelists, it is important that children become involved in the interventions that affect them. The importance of children's views is captured in the excerpts from the panelists quoted below:

“This cannot be done without children's perspectives and views and without involving them as part of the solution in the immediate and recovery phase of this pandemic… Children are experts because they know better than us and we need to listen to them”

(Panelist 1—UN Special Representative) “And it takes a very determined effort by the state, by the community and the involvement of children to really address issues of violence”

(Panelist 3—Director of a Children's Legal Center)

## Discussion

### Role of the socioecological model in mapping intervention levels

The role of the social and physical environment in public health approaches underscores the usefulness of the socioecological model in understanding the different levels of interventions to address VAC. Evidence shows that the social and physical environment can introduce vulnerabilities that increase the risk of children to violence (29, 30). Therefore, the social and physical environments are significant determinants of VAC. The socioecological model indicates the interconnectedness of the levels of intervention. Determining these levels is key to the formation of holistic interventions that leverage global, regional, national, communal, familial and individual factors that support interventions to address VAC.

Global level interventions addressing VAC are instrumental in Qatar for the purpose of establishing collaborations with global institutions (31) and committing to global charters (32). Like other countries that have been working under a global umbrella on addressing VAC, Qatar is a signatory to various conventions such as the Convention on the Rights of the Child (CRC), Declaration on the Elimination of Violence Against Women, and the Convention on the Rights of Persons with Disabilities. Most recently, Sidra Medicine's Child Advocacy Team has signed a partnership agreement with the International Society for the Prevention of Child Abuse and Neglect.

Globally-developed frameworks, such as INSPIRE (11) have been useful in mapping and developing interventions to address VAC across the world, including Qatar. The WHO's commitment to supporting countries in implementing INSPIRE shows the role of global bodies in supporting national efforts. This shows the role of global frameworks in designing and evaluating national interventions. Further, global frameworks enable the standardization of responses by marking key areas of concern, enabling both national evaluations and inter-country comparisons. Specifically, for evaluations, global prevalence has been used to estimate the status of Qatar and other Arab countries by comparing national to global data (33).

WHO's (6) Global status report on violence prevention 2020 showed that Qatar had national action plans addressing child maltreatment, youth violence, interpersonal violence, and sexual violence. Despite the gap in the implementation of policies, Qatar has fully enforced and implemented child maltreatment laws, youth violence laws, and sexual violence laws (**7**). Some significant successes of legislation include eliminating the use of child jockeys as an action against child labor. However, some issues are yet to be addressed with appropriate policies leading to some children being left behind. For instance, the age of criminal responsibility is still relatively low compared to the global definition of a child as a person under the age of 18 years old. Many other gaps exist between the Qatari legislation and policies enshrined in the Convention on the Rights of the Child. Hence, there is a need for the harmonization of the national laws to meet global standards. Other challenges facing implementation and policies in Qatar include slow development of important legislations (34), the complexity of implementation, lack of moral will, lack of trained implementation human resources (35) and lack of evidence and information due to paucity in data (32, 34, 36). These challenges may hamper the cumulative benefits of policies and interventions. Hence, ratifying and strengthening the laws and introducing a framework that coordinates all the related legislations will be a significant step forwards in addressing VAC in Qatar.

The community and family are crucial enablers and indispensable partners in efforts against VAC. Families should be supported through parenting support interventions and mobilizing communities to create safe spaces for children. The consideration of the family and community settings places families and community members as major stakeholders for community interventions aimed at addressing VAC (37). Hence, collaborative teams should include both parents and community representatives as well as identify community resources that can be used in interventions to end VAC. The findings of a global review by Coore Desai et al. (38), found that parental programs had immense potential in preventing and reducing risk factors to child maltreatment. In addition to reducing abusive parenting, parental programs such as home visitation address other types of family violence that intersect with VAC (37, 39).

Physical environments should be encompassed in the planning of residential areas and schools to ensure that children have play facilities that connect them with friends to avoid social isolation and the development of antisocial tendencies. It is also important that interventions are contextualized to work in the unique social and physical conditions in Qatar (40–42). According to Foody et al. (34), contextualizing interventions enables the development of local frameworks that can mitigate factors inherent within a given context. For instance, it would be essential to consider social and cultural acceptance and parent and community beliefs surrounding the conceptualization of child discipline and child protection in Qatar.

There is an evident need and support for involving children in addressing VAC. Despite the widely recognized importance of child participation, there seem to be challenges in actively involving children in the interventions. There could be myriads of reasons for the passive role of children in the addressing of VAC in Qatar. Firstly, children take the position of the vulnerable and at-risk population that needs protection. This positioning purposely places them in a position that does not require active participation. The CRC reiterates that children have the right to involvement (43). Studies have shown that children have emic perspectives that are useful for fleshing out solutions (44). Their involvement; therefore, provides information and experiences that cannot be obtained from surveys or adult and panelist information (31). The dilemma of engaging children in addressing VAC comes up when the need for child perspectives, that is important, may at the same time expose children to certain risks. This calls for the development of a child participation framework in Qatar that will ensure children's voices are captured in policies and interventions while at the same time they are protected from any adverse outcomes from participation.

### Public health approaches to addressing VAC in Qatar

At the national level, public health approaches that focus on promoting health and wellbeing are manifested in the improvement of healthcare infrastructure and human resources. An example of such efforts includes the National Health Strategy 2018–2022 that puts the health of children and adolescents first in Qatar. The aims of the National Health Strategy include improving nutrition and healthy lifestyles among other positive healthcare changes in children and adolescents. It is now evident that multiple programs seeking to meet specific healthcare needs of the Qatari population are now in place. Of significance to this paper, is the utility of the healthcare systems in addressing VAC and promoting positive health and wellbeing of children.

Firstly, the healthcare system is positioned strategically to identify and refer victims of violence and abuse to specialist departments. This informed the creation of the nationwide Child Protection from Abuse Committee under the Ministry of Public Health in 2018. Hospital and clinical settings play a crucial role in secondary and tertiary prevention of VAC. Secondary prevention involves reoccurrence of violence such as through screening programs, addressing risk factors and referring or alerting other child services, while tertiary prevention involves prevention of death or disability by intervening with physical, psychological, and behavioral difficulties emanating from violence (45, 46). In Qatar, HMC, Sidra Medicine, the Primary Health Care Corporation (PHCC), the Psychiatric Hospital, the Behavioral healthcare Center, and the Naufar Addiction Treatment Center—all play a crucial role in addressing VAC.

Secondly, promoting children's positive health and wellbeing is a dominant finding in this paper and has been coined as positive health approach. The use of the term positive health is purposely meant to lay emphasis on the creation of health and wellbeing rather than the prevention of disease (47). This is a prevailing debate in the public health discipline that has the potential of being utilized to address VAC in Qatar. Public health approaches that ensure children experience optimum health and wellbeing with the mission that healthy children will be at reduced risk to intergenerational violence and will have increased resilience. Lansford et al. (48) refer to this paradigm change as shifting from “child survival to child wellbeing”. According to the Global Strategy for Women's, Children's, and Adolescents' Health 2016–2030 (49), building health and wellbeing of children and adolescents keeps them alive and healthy. Further, it reduces poverty, stimulates economic productivity and growth, and job creation, reducing risks of violence. Qatar has employed specific strategies in health education; supportive parenting; nutrition; immunization; psychosocial support; prevention of injuries, violence, harmful practices and substance abuse; sexual and reproductive health information services; and management of communicable and non-communicable diseases. All these show a focus on positive health and wellbeing of children.

The responsibility of primary prevention of VAC is shouldered across departments, institutions, and ministries. The multifaceted nature of VAC requires a multisectoral approach. The departments involved include education, labor, social services, legal and public health. The involvement of multiple sectors is aimed at covering the myriads of risk factors associated with VAC and forms the basis for the multisectoral approach. However, PAHO (50) reports that a comprehensive response requires that all sectors work together because the individual efforts are complementary and supplementary in their approaches. Major stakeholders from diverse organizations operating on various child issues have come together under the same umbrella in Qatar to work together to end VAC. However, the involvement of disparate sectors is not void of challenges such as duplication of interventions. Therefore, there is an imperative need for a framework to coordinate the diverse sectors and align their goals, coordinate the stakeholders, and mobilize resources toward the common goal of eliminating VAC.

### Study limitation

There is a limitation in this review relating to the use of online public videos. Despite the video of a high-profile panel discussion providing rich and up-to-date information for qualitative research, this source has practical, analytic, methodological, and ethical limitations (22, 51). Firstly, although the researchers' specific research interest does not directly influence speakers in the public video, they are instead influenced by a wider public audience and cameras in the venue, and thereby they may act accordingly, or so-called “reactivity” (22, 51–53).

## Conclusion and recommendations

To conclude, the current approaches used in Qatar to address VAC compare favorably with those deemed by panelists as valuable for mitigating the scourge of VAC. This is evidenced by the multipronged approach used at the national level as well as the country's commitment to global efforts to address VAC. To increase Qatar's readiness to combat the issue of VAC, the nation should enhance the capacity of health institutions to work with other sectors and stakeholders to support the public health approach to preventing VAC and associated risk factors, develop a strategy to effectively manage multisectoral collaborations that are guided by the INSPIRE strategies, and take steps to fill the missing gaps in the framework and interventions. Also, Qatar needs to create a database for documenting reported cases of VAC to support researchers, policymakers and intervention developers to work with up-to-date data. This should come with advanced data collection tools and improved reporting mechanisms.

## Data availability statement

The original contributions presented in the study are included in the article/supplementary material, further inquiries can be directed to the corresponding author/s.

## Author contributions

AA-M and KS contributed to conception and design of the study and wrote the first draft of the manuscript. AA-M performed the transcription of the visual material. KS performed the thematic analysis. SA-H, SA, NJ, and GA-H wrote the methodology section of the manuscript. All authors contributed to manuscript revision, read, and approved the submitted version.

## Funding

The publication of this article was funded by Qatar National Library.

## Conflict of interest

The authors declare that the research was conducted in the absence of any commercial or financial relationships that could be construed as a potential conflict of interest.

## Publisher's note

All claims expressed in this article are solely those of the authors and do not necessarily represent those of their affiliated organizations, or those of the publisher, the editors and the reviewers. Any product that may be evaluated in this article, or claim that may be made by its manufacturer, is not guaranteed or endorsed by the publisher.
